# Testing the causal impact of plasma amyloid on total Tau using a genetically informative sample of adult male twins

**DOI:** 10.1016/j.nbas.2025.100139

**Published:** 2025-05-17

**Authors:** Nathan A. Gillespie, Michael C. Neale, Matthew S. Panizzon, Ruth E. McKenzie, Xin M. Tu, Hong Xian, Chandra A. Reynolds, Michael J. Lyons, Robert A. Rissman, Jeremy A. Elman, Carol Franz, William S. Kremen

**Affiliations:** a Virginia Institute for Psychiatric and Behaviour Genetics, Department of Psychiatry, Virginia Commonwealth University, Box 980126, Richmond, VA 23298-0126, USA; b QIMR Berghofer Medical Research Institute, 300 Herston Road, Herston, Queensland 4006, Australia; c Department of Psychiatry, University of California, San Diego, 9500 Gilman Drive, La Jolla, CA 92093, USA; d Department of Psychology, Boston University, 64 Cummington Mall, Boston, MA 02215, USA; e Winston School of Education and Social Policy at Merrimack College, 315 Turnpike Street, North Andover, MA 01845, USA; f Department of Family Medicine and Public Health, University of California, San Diego, 9500 Gilman Drive, La Jolla, CA 92093, USA; g Department of Epidemiology and Biostatistics, Saint Louis University, 3545 Lafayette Ave, St. Louis, MO 63104, USA; h Research Service, VA St. Louis Healthcare System, 1 Jefferson Barracks Drive, St. Louis, MO 63125, USA; i Institute for Behavioral Genetics and Department of Psychology and Neuroscience, University of Colorado, Boulder, 1480 30th Street, Boulder, CO 80309, USA; j Department of Psychological and Brain Sciences, Boston University, 64 Cummington Mall, Boston, MA 02215, USA; k Sam and Rose Stein Institute for Research on Aging, University of California San Diego, 9500 Gilman Drive, La Jolla, CA 92093, USA

**Keywords:** Cascade hypothesis, Plasma, Amyloid-beta, Tau, Twin, Gene, Direction of causation

## Abstract

The amyloid cascade hypothesis predicts that amyloid-beta (Aβ) aggregation drives tau tangle accumulation. We tested competing causal and non-causal hypotheses regarding the direction of causation between Aβ40 and Aβ42 and total Tau (t-Tau) plasma biomarkers. Plasma Aβ40, Aβ42, t-Tau, and neurofilament light chain (NFL) were measured in 1,035 men (mean = 67.0 years) using Simoa immunoassays. Genetically informative twin modeling tested the direction of causation between Aβs and t-Tau. No clear evidence that Aβ40 or Aβ42 directly causes t-Tau was observed. Instead, the alternative causal hypotheses also fit the data well. In contrast, exploratory analyses suggested a causal impact of the Aβ biomarkers on NFL. Separately, reciprocal causation was observed between t-Tau and NFL. Plasma Aβ40 or Aβ42 do not appear to have a direct causal impact on t-Tau, though our use of total rather than phosphorylated tau was a limitation. In contrast, Aβ biomarkers appeared to causally impact NFL in cognitively unimpaired men in their late 60 s.

According to the amyloid cascade hypothesis [1], amyloid-beta (Aβ) aggregation drives accumulation of tau tangles, resulting in synaptic dysfunction, neurodegeneration and progression to cognitive decline. This implies a causal link from Aβ aggregation to tau tangles. To our knowledge this link has not been empirically tested using genetically informative direction-of-causation modeling.

We previously explored the heritability of blood-based biomarkers related to risk of Alzheimer’s Disease in a population-based sample of early old-age men [2]. Additive genetic influences explained 44% to 52% of the total variances in Aβ42, Aβ40, total tau (t-Tau), and neurofilament light chain (NFL), a marker of neurodegeneration. All remaining variances were explained by non-shared environmental influences. Since Aβ aggregation was best explained by genetic and non-shared environmental influences [2], if either Aβ phenotypically causes t-Tau, then significant genetic *and* environment covariance should be observed between Aβ and t-Tau biomarkers. Instead, we found that both Aβ42 and Aβ40 were genetically uncorrelated with t-Tau. This absence of genetic correlation is inconsistent with a direct causal relationship from Aβ to t-Tau under standard direction-of-causation models, as any causal influence would transmit both genetic and environmental effects proportional to their contributions to the causal variable. We did observe a significant environmental correlation between Aβ42 and t-Tau, but a near-zero estimate of the genetic correlation. This pattern is difficult to reconcile with the causal model predicted by the amyloid cascade hypothesis. Given the fundamental importance of the amyloid cascade hypothesis to AD pathogenesis, we believe rigorous empirical testing beyond simple correlation patterns is warranted. Direction of causation modeling provides a more comprehensive statistical approach that can falsify specific causal hypotheses by leveraging the expected patterns in cross-twin cross-trait correlations.

## Aim

Without randomized control trials, Mendelian Randomization or longitudinal data, testing causality between complex traits is difficult. However, by analyzing genetically informative twin data and leveraging the expected differences in the patterns of cross-twin cross-trait correlations, it is possible to falsify hypotheses about the direction of causation between two variables measured on a single occasion [[3], [4], [5], [6], [7]]. Using this approach, we tested competing hypotheses regarding the direction of causation between Aβ and t-Tau plasma biomarkers. We also included exploratory analyses modelling the direction of causation between Aβ and NFL, and between t-Tau and NFL.

## Methods

### Subjects

Detailed demographic characteristics of this sample were described in our previous publication [2]. Additional demographic information based on the VETSA wave 3 assessment is provided in the Supplementary Materials and Table S1. The present study comprised of men from the Vietnam Era Twin Study of Aging (VETSA) who participated in a third assessment wave (mean age = 68.2, SD = 2.5, range = 61.4 to 73.3) when plasma biomarkers were examined [2].

### Blood-based biomarker data

Blood was collected under fasting conditions before acquisition and storage at −80 °C. The Simoa Human Neurology 3-plex A (N3PA) Immunoassay was used to measure Aβ40, Aβ42, and t-Tau, while the Simoa NF-light assay was used to measure NFL (Quanterix™, Billerica, MA, USA). Biomarkers were regressed onto age at assessment, testing site, storage time, self-reported race/ethnicity, and whether or not twins pairs were assessed on the same day. Residual scores were calculated using the umx_residualize function [8]. Next, the data were normal ranked in R_4.0.3_ [9] and absolute values greater than three standard deviations (SDs) were eliminated to reduce skew. This eliminated a total of 12, 8, 12 and 15 subjects with Aβ40, Aβ42, NFL and t-Tau data respectively. Depending on the biomarker, there were between 988 and 1035 individuals (58 % monozygotic and 42 % dizygotic twins) with complete data.

Individual Aβ40 and Aβ42 biomarkers were analyzed both separately and in combination, rather than as a ratio. This approach was chosen based on our previous findings [2] regarding statistical concerns with the Aβ42/Aβ40 ratio. While the primary focus of our study was testing the amyloid cascade hypothesis regarding Aβ and t-Tau, we also explored relationships with NFL, a marker of large-caliber axonal degeneration. We hypothesized that if the amyloid cascade hypothesis is correct, Aβ aggregation might directly impact neurodegeneration (as measured by NFL) either independently or through tau-mediated processes.

### Statistical analyses

Based on biometrical genetic methods [10], the OpenMx_2.20.6_ software package [11] with the raw data Full Information Maximum Likelihood (FIML) option and NPSOL optimizer in R_4.2.2_ [9] was used to decompose the total variance in each biomarker into latent additive genetic (A), shared or common (C) environment, and non-shared or unique (E) environmental influences (which represent unique experiences and measurement error) while testing competing causal and non-causal hypotheses. Further details on the estimation of these components are provided in the Supplement, along with Supplementary Fig. S1, which illustrates the theoretical model. Briefly, direction of causation modeling works by comparing the fit of statistical models that represent different causal scenarios. When two traits have different heritability patterns, these models can distinguish which causal direction best explains the observed cross-twin cross-trait correlations (where one twin's trait A is correlated with the co-twin's trait B). In the context of the amyloid cascade hypothesis, plasma Aβ are considered proxies for amyloid accumulation, t-Tau primarily reflects neuronal injury and neurodegeneration rather than specifically measuring phosphorylated tau that is the primary component of neurofibrillary tangle pathology, and NFL indicates axonal degeneration.

We note that although our previous analyses supported AE models for these biomarkers [2], we initially fit ACE models here to maximize our ability to detect differences between competing causal hypotheses. By specifying all three sources of variance (A, C, and E), we increase the likelihood that the expected variance-covariance patterns will differ across causal models, thereby enhancing our power to discriminate between alternative causal hypotheses. This approach provides a more rigorous test of the direction of causation than would be possible with more constrained AE models.

**Fig. 1:**
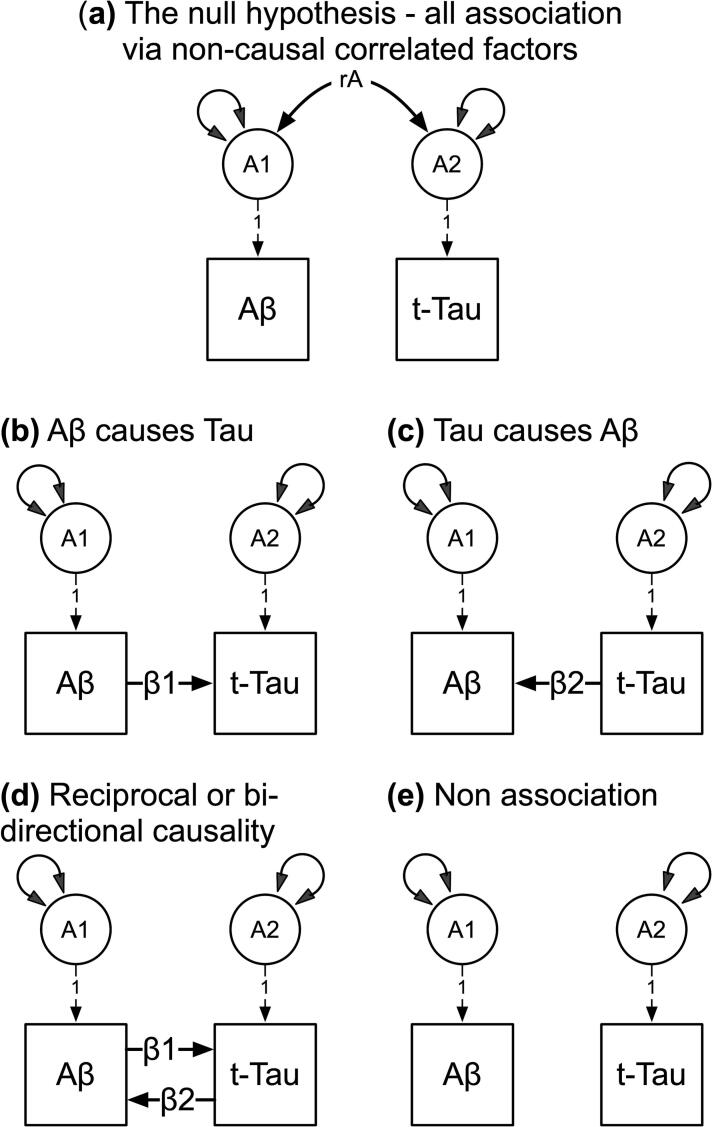
Competing hypothetical models to account for the association between the Aβ and t-Tau biomarkers. A1 and A2 are latent additive genetic influences for the Aβ and t-Tau plasma biomarkers respectively. Double-headed arrows denote the A1 and A2 genetic variances, and non-causal additive covariance (rA). Latent environmental influences, including residuals and means not illustrated for brevity. Competing models: (a) non-causal association stemming from correlated additive genetic influences (rA); (b) uni-directional Aβ causes t-Tau via the regression coefficient β1; (c) uni-directional t-Tau causes Aβ via the regression coefficient β2; (d) reciprocal or bi-directional causation between Aβ and t-Tau; and (e) no association between Aβ and t-Tau where β1 = β2 = 0.

Illustrated in Fig. 1, our null hypothesis predicted that associations between the Aβ and t-Tau biomarkers were explained by correlated, non-causal genetic and environmental influences (for brevity, only genetic influences are shown). We analyzed Aβ40 and Aβ42 separately and tested four competing, nested hypotheses: (**b**) Aβ causes t-Tau via β_1_; (**c**) t-Tau causes Aβ via β_2_; (**d**) reciprocal causation between Aβ and t-Tau via β_1_ and β_2_; and (**e**) no association i.e. β_1 =_ β_2_ = 0.

We also modelled the joint impact of both Aβs on t-Tau (see Supplementary Fig. S2, showing competing uni-directional causal pathways between Aβs and t-Tau), followed by exploratory causal modelling between the Aβs and NFL, and finally between t-Tau and NFL. The joint analysis of both Aβ40 and Aβ42 with t-Tau provides additional information by examining their combined influence on t-Tau. This multivariate approach captures more variance in amyloid pathology than either marker alone and tests whether the combined amyloid signal shows a causal relationship with tau that might not be evident when testing each isoform separately.

The goodness of fit for each model was determined using the likelihood ratio statistic, which is the change in the minus two log-likelihood between the null and each competing model. This statistic, Δ-2LL, is asymptotically distributed as chi-squared with degrees of freedom equal to the difference in the number of free parameters between the null and each competing model. Our determination of the best-fitting model was also based on the optimal balance of complexity and explanatory power using Akaike’s Information Criterion (AIC) [12].

## Results

Model fit comparisons are shown in Table 1. For each set of analyses (i, ii & iii) the null hypothesis predicted that any observed association between Aβ and Tau was attributable to non-causal, correlated genetic and environmental factors.

**Table 1:** Multivariate model fitting comparisons between the non-causal correlated factors reference model (a) and the two causal (b-c), bi-directional or reciprocal causation (d), and the no association (e) models.

(i) Aβ40 & Tau	ep	−2LL	df	Δ-2LL	Δdf	p	AIC
(a) Correlated / non-causal	11	4482.91	1930				4504.91
(b) Aβ40 **→** Tau	9	4484.34	1932	1.43	2	0.4895	4502.34
(c) Tau → Aβ40	9	4484.36	1932	1.45	2	0.4842	4502.36
(d) Reciprocal causation	10	4483.36	1931	0.45	1	0.5013	4503.36
(e) No association	8	4484.47	1933	1.55	3	0.6699	4500.47
**(ii) Aβ42 & Tau**							
(a) Correlated / non-causal	11	4423.18	1917				4445.18
(b) Aβ42 **→** Tau	9	4424.15	1919	0.98	2	0.6139	4442.15
(c) Tau → Aβ42	9	4423.65	1919	0.47	2	0.7891	4441.65
(d) Reciprocal causation	10	4423.35	1918	0.17	1	0.6775	4443.35
(e) No association	8	4439.72	1920	16.54	3	0.0009	4455.72
**(iii) Both Aβs & Tau**							
(a) Correlated / non-causal	21	6120.15	2910				6162.15
(b) Aβs **→** Tau	17	6123.54	2914	3.39	4	0.4946	6157.54
(c) Tau → Aβs	17	6122.68	2914	2.53	4	0.6390	6156.68
(d) Reciprocal causation	19	6120.86	2912	0.71	2	0.7003	6158.86
(e) No association	15	6157.59	2916	37.44	6	0.0000	6187.59

ep = number of estimated parameters, −2LL = −2 x log-likelihood, Δ-2LL = change in −2 x log-likelihood, Δ df = change in degrees of freedom, AIC = Akaike Information Criteria. In each of the three analyses (i, ii & iii), the nested sub-models (b, c & d) each provided good fits to the data in terms of non-significant Δ-2LL & lower AIC values when compared to the null.

When testing the ‘Aβ40 causes Tau’ hypothesis, all four competing hypotheses (both uni-directional, the reciprocal, and the no association model) provided a good fit to the data in terms of non-significant changes in chi-square, whereas the ‘no association’ hypothesis provided the lowest AIC.

When testing the ‘Aβ42 causes Tau’ hypothesis, the changes in chi-square for all three competing causal hypotheses were non-significant, whereas the AIC was lowest for the ‘Tau causes Aβ42′ hypothesis. Note that the ‘no association’ hypothesis could be rejected based on the significant change in chi-square and higher AIC value relative to the three other competing hypotheses.

When testing the ‘Aβ40 & Aβ42 (combined) cause Tau’ hypothesis, the changes in chi-square for each of the three causal hypotheses were again non-significant. However, very little separated their corresponding AIC values. Note that the ‘no association’ hypothesis could again be rejected in terms of the significant chi-square change and highest AIC.

In exploratory analyses, we modelled the multivariate impact of Aβ40 and Aβ42 (Aβs) on NFL (Supplementary Table S2). Among the competing hypotheses, the uni-directional ‘Aβs cause NFL’ hypothesis provided a marginally better fit to the data as judged by the non-significant change in chi-square and lowest AIC. Finally, when modelling the association between t-Tau and NFL, the reciprocal causation model provided a (marginally) best fit to the data, followed next by the ‘t-Tau causes NFL’ hypothesis.

## Discussion

To our knowledge, this is the first genetically informative test of the direction of causation between blood-based biomarkers related to Alzheimer’s Disease. We found no unequivocal support for a causal impact of either Aβ40 or Aβ42 on t-Tau. Instead, alternative uni-directional and reciprocal hypotheses provided comparable fits to the data. In contrast, exploratory analyses suggest a causal impact of both blood-based Aβ biomarkers on NFL, and a reciprocal causal association between t-Tau and NFL.

A key limitation of the Direction of Causation modeling approach is its reduced power to differentiate between causal directions when traits have similar heritability patterns. In our previous work [2], all biomarkers showed similar genetic (44–52 %) and environmental contributions, which likely contributes to our inability to definitively distinguish between alternative causal hypotheses. In such cases, even our relatively large sample of over 1,000 individuals may be insufficient for definitive testing of competing causal hypotheses. This limitation explains why we were able to exclude the 'no association' hypothesis but could not clearly differentiate between the remaining causal models.

The absence of clear, empirical support for a causal impact of Aβ on t-Tau, which would be consistent with the amyloid-beta cascade hypothesis, should be interpreted in the context of five additional considerations. First, our study included only men. This limitation is important given that sex differences in AD biomarkers and pathology have been reported. Future studies should examine these causal relationships in more diverse populations including females. Second, our sample was predominately cognitively unimpaired. The proportion of men with mild cognitive impairment (MCI) was 15 %. Causal signals may emerge as the sample ages and the prevalence of MCI increases over time. Third, we relied on plasma biomarkers. While accessible, affordable, and heritable [2], we note that dilution, degradation, and metabolism may introduce variation unrelated to AD-related brain changes. This may limit the predictive validity of these plasma biomarkers to model causation. Ultrasensitive immunoassays and novel mass spectrometry techniques that attempt to address this limitation have begun to show promise in terms of better plasma biomarker measurement [[13], [14]]. These two limitations are underscored by Coomans et al. [15] who analyzed data from a very small sample of older monozygotic twins with a relatively large number of APOE-ε4 carriers and found significant associations between Aβ-PET and tau-PET. Fourth, to the extent that plasma Aβ is brain derived, it may nevertheless reflect general health conditions rather than brain amyloid accumulation. Finally, we relied on total tau rather than phosphorylated Tau (p-Tau), which aggregates into neurofibrillary tangles and is therefore likely to be a more relevant indicator of AD pathogenic processes. Indeed, the p-Tau 181, 217 and 231 isoforms have been shown to predict amyloidosis and progression to AD [16]. The genetic variance of these isoforms remains undetermined (including their covariance and direction of causation) with the Aβ and NFL biomarkers. Unlike our results for t-Tau, it is plausible that direction of causation modeling with p-Tau isoforms might be consistent with the amyloid cascade hypothesis.

Our findings should not be interpreted as definitively refuting the amyloid cascade hypothesis. Nonetheless, they have important implications for both theoretical models of AD pathogenesis and the validation of blood-based biomarkers. The absence of clear causal relationships between plasma Aβ and t-Tau alongside the observed causal impact of Aβ on NFL suggests more complex relationships between biomarkers. Specifically, results may align with the hypothesis when considering that the amyloid-neurodegeneration relationship depends on the presence and types of abnormal tau accumulation.

We acknowledge that our use of t-Tau, which reflects general neurodegeneration rather than specific tau pathology, limits strong conclusions about the amyloid-tau relationship. As blood-based biomarkers advance toward clinical and consumer applications, this natural experimental approach provides a valuable methodological template in lieu of RCTs for evaluating newer, more specific biomarkers such as p-Tau isoforms. Future studies applying twin-based causal modeling will be crucial for properly evaluating the construct validity of plasma biomarkers and refining our understanding of AD pathogenesis. This approach offers a powerful tool for testing whether biomarkers reflect the causal relationships they are presumed to represent before their widespread implementation in clinical or direct-to-consumer contexts.

Notwithstanding the absence of a population-based same-age replication sample, to the extent that that plasma biomarkers are considered informative peripheral indicators of prodromal AD [[16], [17], [18]], our analyses suggest little evidence for a causal impact of Aβ40 or Aβ42 on t-Tau, when based on community-dwelling sample of men in their late 60 s.

## Funding sources

This work was supported by the 10.13039/100000049National Institute on Aging at the 10.13039/100000002National Institutes of Health grant numbers R01s AG050595, AG022381, AG037985; R25 AG043364, F31 AG064834; P01 AG055367, AG062483; and K01 AG063805. The funding sources had no role in the preparation, review, or approval of the manuscript, or the decision to submit the manuscript for publication. The content of this manuscript is solely the responsibility of the authors and does not necessarily represent the official views of the NIA/NIH, or the VA.

## Declaration of generative AI in scientific writing

Nothing to declare.

## CRediT authorship contribution statement

**Nathan A. Gillespie:** Writing – review & editing, Writing – original draft, Methodology, Investigation, Formal analysis, Conceptualization. **Michael C. Neale:** Writing – review & editing, Software, Methodology. **Matthew S. Panizzon:** Writing – review & editing, Data curation. **Ruth E. McKenzie:** Writing – review & editing. **Xin M. Tu:** Writing – review & editing. **Hong Xian:** Writing – review & editing. **Chandra A. Reynolds:** Writing – review & editing. **Michael J. Lyons:** Writing – review & editing, Funding acquisition. **Robert A. Rissman:** Writing – review & editing, Data curation. **Jeremy A. Elman:** Writing – review & editing, Data curation. **Carol Franz:** Writing – review & editing, Funding acquisition, Data curation. **William S. Kremen:** Writing – review & editing, Funding acquisition, Data curation.

## Declaration of competing interest

The authors declare that they have no known competing financial interests or personal relationships that could have appeared to influence the work reported in this paper.

## Data Availability

Data are publicly available through requests at the VETSA website (http://www.vetsatwins.org).
