# Supplementary material for: Testing the causal impact of plasma amyloid on total Tau using a genetically informative sample of adult male twins
Source: Aging Brain. 2025 May 17;7:100139. doi: 10.1016/j.nbas.2025.100139 (PMC12145560; doi:10.1016/j.nbas.2025.100139)
Supplement: Supplementary Data 1 [file mmc1.docx]

# Supplement

The third wave of data collection (VETSA 3) comprised male twins from the Vietnam Era Twin Registry with a mean age of 68.2 years (SD=2.5, range=61.4-73.3). This community-dwelling cohort represents American men in their age range, with participants residing throughout the United States. As documented elsewhere (Kremen, Beck, et al., 2019; Kremen, Franz, et al., 2019), approximately 80% reported no combat experience during military service. The sample included both monozygotic (58%) and dizygotic (42%) twin pairs, with approximately 15% meeting criteria for mild cognitive impairment at VETSA 3. The sociodemographic profile of this cohort makes it well-suited for population-based inference (Kremen, Franz, et al., 2019).

**Table S1.** Demographic Characteristics of the VETSA Sample.

| **Characteristic** | **Value** |
| --- | --- |
| Age at assessment (years), mean (SD), range | 68.2 (2.5), 61.4-73.3 |
| Sample Size by Zygosity (%): |  |
| - Monozygotic pairs | ~58% |
| - Dizygotic pairs | ~42% |
| Lifetime education (%): |  |
| - Completed high-school | 97% |
| - Some college education | 60% |
| - Bachelor's degree or higher | 29% |
| Military combat exposure (%): |  |
| - No combat experience | ~80% |
| - Some combat experience | ~20% |
| Cognitive status (%): |  |
| - Cognitively normal | ~85% |
| - Mild Cognitive Impairment | ~15% |
| Race/ethnicity (%): |  |
| - Caucasian | 88.3% |
| - Black | 5.3% |
| - Hispanic | 3.4% |
| - Other | 3.0% |
| Note: Values based on Kremen et al. (Kremen, Beck, et al., 2019; Kremen, Franz, et al., 2019). Number of complete and incomplete twin pairs by zygosity and biomarker reported in Gillespie et al. (Gillespie et al., 2023). The total sample size for each biomarker ranges from 988 to 1035 individuals, depending on the specific biomarker being analyzed. Values marked with '~' are approximations due to slight variations in percentages across different sample sizes. | |

**Statistical analyses**

We applied the Classical Twin Design (CTD), which relies on twins reared together, to decompose the total variation in each plasma biomarker into latent additive (A) genetic variance, shared or common environmental (C), and non-shared or unique (E) environmental variance components. This approach follows standard biometrical genetic methods (Falconer, 1960; Jinks & Fulker, 1970; Martin & Eaves, 1977; Eaves et al., 1978; Neale & Cardon, 1992).

This decomposition is achieved using structural equation modeling (SEM), which partitions the observed variance-covariance structure into these latent A, C, and E components by exploiting the expected genetic and environmental correlations between MZ and DZ twin pairs; MZ twin pairs are genetically identical, whereas DZ twin pairs share, on average, only half of their genes. Therefore, MZ and DZ twin pair correlations (rA) for additive genetic effects are fixed to 1.0 and 0.5 respectively. The CTD assumes no genotype by environmental interactions or correlations, and random parental mating. It also assumes equal shared environmental effects for MZ and DZ twin pairs, including equality of parental treatment, environmental exposure, and no effects caused by placentation [3]. Given this equal environment assumption, the MZ and DZ twin pair correlations (rC) for shared environmental influences are each fixed to 1.0. All non-shared environmental influences (E), including measurement error, are by definition uncorrelated, so the MZ and DZ twin pair correlation (rE) for these 'E' influences is fixed to zero.

This univariate approach can be readily extended to the multivariate case to estimate the size and significance of genetic and environmental influences within and between two or more complex traits, including direction of causation.

Historically, assessing evidence of causality has been challenging without double-blind random case-control experiments or longitudinal designs. In the absence of such data, determining whether an observed association is causal or stems from a correlated liability (where cross-sectional or longitudinal phenotypic associations arise due to correlated, unmeasured background genetic or environmental effects) is difficult. As an alternative to costly genetically informative longitudinal data, we applied an innovative statistical method: direction of causation modeling on cross-sectional, genetically informative data (Hill, 1965; Heath et al., 1993). This approach, which has been applied to various complex behavioral phenotypes (Duffy & Martin, 1994; Neale et al., 1994; Gillespie et al., 2003), requires several key assumptions (Heath et al., 1993):

1. Members of a twin pair do not have any mutual effect on one another (i.e., no sibling cooperation/rivalry), either within or across variables.
2. The relationship between variables is equivalent for twin 1 and twin 2.
3. Twin pair correlations differ between the variables being studied [7].
4. There are no unmeasured variables influencing both measures, which could inflate correlations arising through the causal influence of one variable on the other.

If these assumptions are satisfied, differences in the patterns of cross-twin cross-plasma biomarker correlations can allow us to falsify strong hypotheses about the direction of causation between two variables measured on a single occasion. The power to do this increases when there are differences in the causes of variation in one biomarker versus another (Heath et al., 1993).

**Supplementary Figure S1.** Theoretical model for modeling direction of causation using genetically informative twin data.


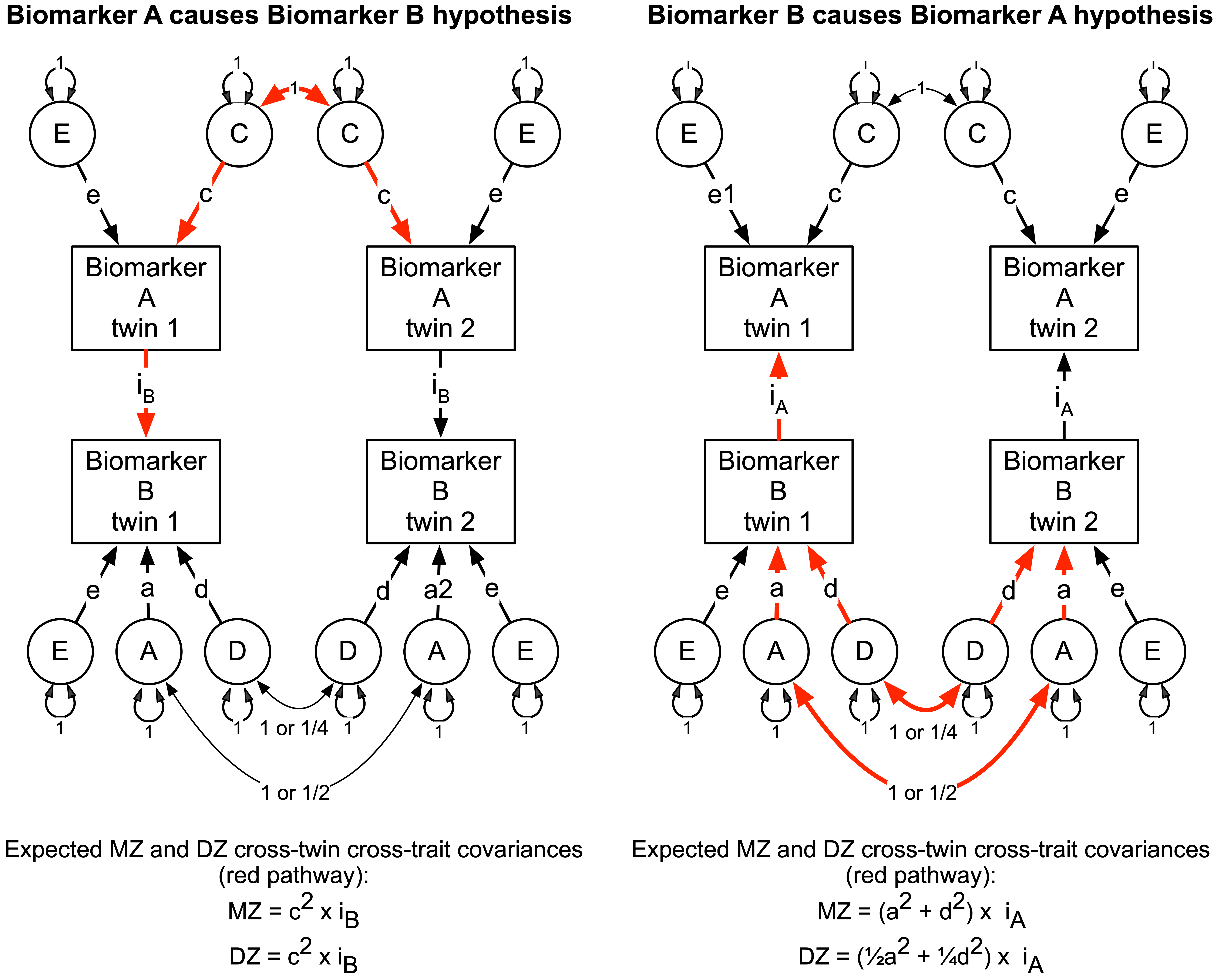


Figure S1 illustrates this approach. Assuming variable A is best explained by shared (C) and non-shared (E) environmental effects, while variable B is best explained by additive genetic (A), dominant genetic (D), and non-shared (E) environmental effects, we can use Wright's (Wright, 1934) path tracing rules. The 'A-to-B' and 'B-to-A' hypotheses generate different expected monozygotic and dizygotic cross-twin cross-biomarker correlations (e.g., correlation between Twin 1 Biomarker A and Twin 2 Biomarker B), whose goodness of fit can be compared using likelihood-ratio chi-squared tests.

In the Classical Twin Design of twins reared together, the effects of non-additivity or dominance (D) and common environmental (C) influences are negatively confounded, and therefore, cannot be modelled simultaneously (Martin et al., 1978). Since the sample sizes required to detect 'D' as a source of variation are very large, even for variables measured on a continuous liability scale, we chose to model 'C' influences in all subsequent univariate and multivariate models.

**Supplementary Figure S2**. Expected cross-twin cross-trait covariances for monozygotic (MZ) and dizygotic (DZ) twin pairs under the competing uni-directional hypotheses: A causes B; and B causes A.


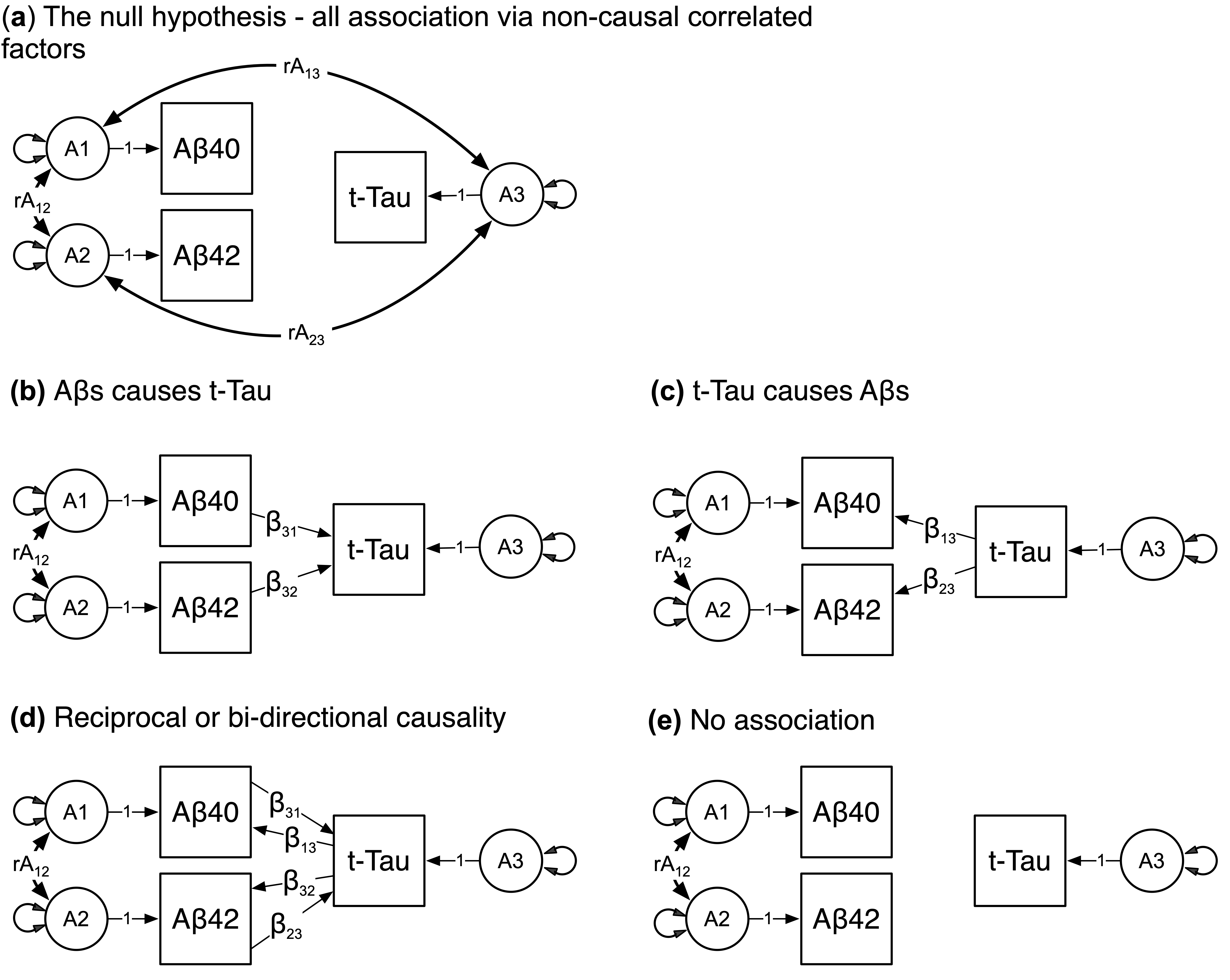


**Footnote**: A1, A2, and A3 refer to the latent additive genetic influences for the Aβ40, Aβ42, and t-Tau biomarkers respectively. Double-headed arrows denote the variances and the non-causal additive genetic covariances. Latent environmental influences, residuals, and means not shown for brevity. Competing models: (a) non-causal association stems from correlated additive genetic influences (rA13 & rA23); (b) uni-directional hypothesis where Aβs cause t-Tau via the regression coefficients β31 & β32; (c) uni-directional hypothesis where t-Tau causes Aβs via the regression coefficients β13 & β23; (d) reciprocal or bi-directional causation between the Aβs and t-Tau; and (e) no association between the Aβs and t-Tau.

**Supplementary Table S2**. Model fitting analysis: Impact of Aβ proteins (Aβ40 and Aβ42) on NFL - comparing correlation, causation, and independence or no association models.

| **(i) Both Aβs & NFL** | **ep** | **-2LL** | **df** | **Δ-2LL** | **Δdf** | **p** | **AIC** |
| --- | --- | --- | --- | --- | --- | --- | --- |
| (a) Correlated / non-causal | 21 | 6420.05 | 3003 |  |  |  | 6462.05 |
| (b) Aβs **→** NFL | 17 | 6423.97 | 3007 | 3.92 | 4 | 0.4167 | 6457.97 |
| (c) NFL **→** Aβs | 17 | 6425.97 | 3007 | 5.92 | 4 | 0.2051 | 6459.97 |
| (d) Reciprocal causation | 19 | 6421.17 | 3005 | 1.13 | 2 | 0.5696 | 6459.17 |
| (e) No association | 15 | 6537.34 | 3009 | 117.30 | 6 | 0.0000 | 6567.34 |
|  |  |  |  |  |  |  |  |
| **(ii) NFL & Tau** |  |  |  |  |  |  |  |
| (a) Correlated / non-causal | 11 | 4109.36 | 1960 |  |  |  | 4131.36 |
| (b) NFL **→** Tau | 9 | 4115.62 | 1962 | 6.26 | 2 | 0.0438 | 4133.62 |
| (c) Tau → NFL | 9 | 4113.91 | 1962 | 4.55 | 2 | 0.1029 | 4131.91 |
| (d) Reciprocal causation | 10 | 4110.15 | 1961 | 0.79 | 1 | 0.3736 | 4130.15 |
| (e) No association | 8 | 4141.64 | 1963 | 32.28 | 3 | 0.0000 | 4157.64 |

**Footnote**: ep = number of estimated parameters, -2LL = -2 x log-likelihood, Δ-2LL = change in -2 x log-likelihood, Δ df = change in degrees of freedom, AIC = Akaike Information Criteria. In each of the three analyses (i, ii & iii), the nested sub-models (b, c & d) each provided good fits to the data in terms of non-significant Δ-2LL & lower AIC values when compared to the null.

References

Wright, S. (1934). The method of path coefficients. *Annals of Mathematical Statistics*, *5*, 161-215.

Falconer, D. S. (1960). *Introduction to Quantitative Genetics*. Oliver & Boyd.

Hill, A. B. (1965). The Environment and Disease: Association or Causation? *Proceedings of the Royal Society of Medicine*, *58*, 295-300. <http://www.ncbi.nlm.nih.gov/pubmed/14283879>

Jinks, J. L., & Fulker, D. W. (1970). Comparison of the biometrical genetical, MAVA, and classical approaches to the analysis of human behavior. *Psychological Bulletin*, *73*(5), 311-349.

Martin, N. G., & Eaves, L. J. (1977). The genetical analysis of covariance structure. *Heredity*, *38*(1), 79-95.

Eaves, L. J., Last, K. A., Young, P. A., & Martin, N. G. (1978). Model-fitting approaches to the analysis of human behaviour. *Heredity*, *41*(2), 249-320.

Martin, N. G., Eaves, L. J., Kearsey, M. J., & Davies, P. (1978). The power of the classical twin study. *Heredity (Edinb)*, *40*(1), 97-116. (MEDLINE)

Neale, M. C., & Cardon, L. R. (1992). *Methodology for Genetic Studies of Twins and Families* (1st ed.). Kluwer Academic Publishers.

Heath, A. C., Kessler, R. C., Neale, M. C., Hewitt, J. K., Eaves, L. J., & Kendler, K. S. (1993). Testing Hypotheses About Direction of Causation Using Cross-Sectional Family Data. *Behavior Genetics*, *23*(1), 29-50. <https://doi.org/Doi> 10.1007/Bf01067552

Duffy, D. L., & Martin, N. G. (1994). Inferring the direction of causation in cross-sectional twin data: theoretical and empirical considerations [see comments]. *Genetic Epidemiology*, *11*(6), 483-502.

Neale, M. C., Walters, E., Health, A. C., Kessler, R. C., Perusse, D., Eaves, L. J., & Kendler, K. S. (1994). Depression and parental bonding: cause, consequence, or genetic covariance? *Genetic Epidemiology*, *11*(6), 503-522. <https://doi.org/10.1002/gepi.1370110607>

Gillespie, N. A., Zhu, G., Neale, M. C., Heath, A. C., & Martin, N. G. (2003). Direction of causation modeling between cross-sectional measures of parenting and psychological distress in female twins. *Behavior Genetics*, *33*(4), 383-396. <http://www.ncbi.nlm.nih.gov/pubmed/14574138>

Kremen, W. S., Beck, A., Elman, J. A., Gustavson, D. E., Reynolds, C. A., Tu, X. M., Sanderson-Cimino, M. E., Panizzon, M. S., Vuoksimaa, E., Toomey, R., Fennema-Notestine, C., Hagler, D. J., Jr., Fang, B., Dale, A. M., Lyons, M. J., & Franz, C. E. (2019). Influence of young adult cognitive ability and additional education on later-life cognition. *Proceedings of the National Academy of Sciences of the United States of America*, *116*(6), 2021-2026. <https://doi.org/10.1073/pnas.1811537116>

Kremen, W. S., Franz, C. E., & Lyons, M. J. (2019). Current Status of the Vietnam Era Twin Study of Aging (VETSA). *Twin Res Hum Genet*, *22*(6), 783-787. <https://doi.org/10.1017/thg.2019.125>

Gillespie, N. A., Elman, J. A., McKenzie, R. E., Tu, X. M., Xian, H., Reynolds, C. A., Panizzon, M. S., Lyons, M. J., Eglit, G. M. L., Neale, M. C., Rissman, R., Kremen, W. S., & Franz, C. (2023). The heritability of blood-based biomarkers related to risk of Alzheimer’s Disease in a population-based sample of early old-age men. *Alzheimer’s & Dementia*, *20*(1), 356-365. <https://doi.org/10.1002/alz.13407>

**Supplementary Table S1**. Multivariate model fitting comparisons between the non-causal correlated factors reference model (a), and the two causal (a-b), reciprocal causation (c), and no association (d) models.

**Footnote**: Aβs = amyloid-beta 42 & 42, NFL = neurofilament light chain, ep = number of estimated parameters, -2LL = -2 x log-likelihood, Δ -2LL = change in -2 x log-likelihood, Δ df = change in degrees of freedom, AIC = Akaike Information Criteria.
